# Prevalence of Congenital Malaria in Kisangani, A Stable Malaria Transmission Area in Democratic Republic of the Congo

**DOI:** 10.1155/2020/2176140

**Published:** 2020-02-25

**Authors:** Labama Otuli Noël, Bosenge Nguma Jean-Didier, Maindo Alongo Mike-Antoine, Katenga Bosunga Gedeon, Mbo Mukonkole Jean-Paulin, Losimba Likwela Joris, Manga Okenge Jean-Pascal

**Affiliations:** ^1^Obstetrics and Gynecology Department, Faculty of Medicine and Pharmacy, University of Kisangani, Kisangani, Democratic Republic of the Congo; ^2^Internal Medical Department, Faculty of Medicine and Pharmacy, University of Kisangani, Kisangani, Democratic Republic of the Congo; ^3^Public Health Department, Faculty of Medicine and Pharmacy, University of Kisangani, Kisangani, Democratic Republic of the Congo

## Abstract

**Background:**

Gestational malaria is a major public health problem. It produces fetal complications such as low birth weight, perinatal mortality, and congenital malaria. The present study is aimed at determining the prevalence of congenital malaria and its neonatal complications in the city of Kisangani.

**Methods:**

We conducted a cross-sectional study in Kisangani from 1 January to 30 September 2018. Our study population was composed of 1248 newborns born in our study sites, during the period of our study. Just after their birth, we performed the thick drop smear in the placental print and in umbilical blood smear.

**Results:**

The prevalence of congenital malaria was 13.98%; 69.23% of newborns who contracted congenital malaria were from 18- to 34-year-old mothers, 53.85% from primiparous mothers, 92.31% from mothers who took intermittent preventive treatment in pregnancy with Sulfadoxine-Pyrimethamine, all (100%) from mothers using the insecticide-treated mosquito nets and 7.69% from HIV-positive mothers. Low birth weight and perinatal mortality were recorded in 76.92% and 7.69% of congenital malaria cases, respectively. Intermittent preventive treatment in pregnancy with Sulfadoxine-Pyrimethamine had no effect on congenital malaria (FE = 0.5218; OR: 0.8, 95% CI: 0.1651-3.8769) and on low birth weight (FE = 0.3675; OR: 1.2308, 95% CI: 0.0037-0.1464); however, it seemed to have protective effect against perinatal mortality (FE = 0.0001; OR: 0.0233, 95% CI: 0.0037-0.1464).

**Conclusion:**

Congenital malaria remains a major problem in stable malaria transmission area like Kisangani, and it is grafted by major perinatal complications, particularly low birth weight and perinatal mortality. We recommend an extended study to clarify the relationship between the outcome of pregnancy and the intermittent preventive treatment in pregnancy with Sulfadoxine-Pyrimethamine.

## 1. Background

Malaria, known since antiquity, remains a public health problem. In the world, in 2010, the population likely to be infected by parasite and to develop disease was 3.2 billion, and the risk was high (more than one out of 1000 chance to contract malaria over a year) for 1.2 billion people [1]. After an unprecedented period of success in the world fight against malaria, the World Health Organization (WHO) reported that malaria-related mortality had remained the same from 2014 to 2016. In 2016, 216 million cases of malaria were reported in 91 countries, an increase of 5 million over the previous year. The number of related deaths has reached 445000, almost like in 2015 [2].

According to the WHO, the African region accounts for 90% of malaria cases and related deaths worldwide [2]. Children under five and pregnant women are the main victims [3, 4].

Every year in Africa, more than 30 million pregnant women live in malaria-endemic areas. The prevalence of malaria during pregnancy is variable in endemic area. It varies from 5 to 40% depending on the country [5]. Malaria infestation of pregnant women is a major public health problem, as this disease is a threat to themselves and their children, with up to 200000 stillborn babies each year due to the presence of malaria during pregnancy. In addition to perinatal mortality, it also causes low birth weight and congenital malaria [2].

The Democratic Republic of Congo (DRC) is the second sub-Saharan African country most affected by malaria after Nigeria. These two countries alone, added to India, account for 40% of malaria cases [6]. In northeast DRC where Tshopo province is located, malaria is the cause of consultation in 37% of all pathologies and the cause of death at 30% of cases [4, 5]. The equatorial facies which dominates this province in general and the city of Kisangani in particular and the presence of Plasmodium falciparum which ensures intense and permanent transmission as well as the insanitary condition of the environment would be the cause.

According to the DRC National Malaria Control Program (NMCP), some factors, including the late and irregular attendance of prenatal consultations (ANC) by pregnant women and the shortage of stock of Sulfadoxine-Pyrimethamine in healthcare settings, make it difficult to control malaria among pregnant women in the DRC [7].

These factors make the prevalence of gestational malaria high in Kisangani, a stable malaria transmission area. In areas of stable transmission of malaria, ovular complications predominate [8]. Therefore, the risk of congenital malaria would not be excluded.

In conducting this study, we set ourselves the goal of raising the prevalence of congenital malaria and its complications in Kisangani City.

## 2. Methods

### 2.1. Study Sites

This study was conducted in the General Reference Hospitals of Kabondo, Lubunga, and Makiso-Kisangani and in the Reference Healthcare Centers Foyer, Matete, and Saint Joseph: all located in the city of Kisangani, a malaria-endemic area in northeastern DRC. These first-level medical structures were selected on the basis of their high attendance and the representativeness of the communes in the city of Kisangani. However, Kisangani commune was not represented because it does not have any first-level hospitals. Thus, we selected General Reference Hospital Kabondo, a second medical training in the commune of Makiso which, for reasons of proximity, receives most of the women residing in Kisangani commune.

### 2.2. Type and Study Period

It is a cross-sectional study from 1 January to 30 September 2018.

### 2.3. Sample Size and Study Population

The study population was composed of all newborns who were born in our study sites, during the period of our study.

Newborns from parturients living in and around Kisangani (less than 30 km) at least 14 days before delivery and without history of taking antimalarial drugs during the same period were included in the study.

To calculate the minimum size of our sample, we used the STATCALC software Epi info 7.2.2.6. Based on the 14.8% of placental malaria cases found in a pilot study conducted by Lukuka et al. [9] in 4 maternity wards in the city of Kinshasa (DRC) which has the same epidemiological aspect as the city of Kisangani, with 95% confidence level and an acceptable 5% margin of error, we found a minimum sample size of 194 cases. For our study, 1248 parturients, who complied with the inclusion criteria, were recruited consecutively. There were no twins among their newborns.

### 2.4. Data Collection

Data collection was prospective. It was done when the mothers of our babies surveyed came to give birth. To collect the data for this study, we used a previously established data collection form. At the admission of parturients to the labor room, the investigators (nurses or midwives) proceeded to interview the parturients. During the interview, the investigators obtained their informed consent for the participation in the study, the attribution of a unique code to each parturient and investigations, related to their socio-anthropometric parameters (age, education level, occupation, and common residence), their background and preventive measures against malaria, in particular, using of the insecticide-treated mosquito nets (ITNs) and intermittent preventive treatment in pregnancy with Sulfadoxine-Pyrimethamine (IPTp-sp). The code assigned to each parturient included the initial of her healthcare structure and the initial of her arrival order at the delivery facility. The same parturient code was attributed after delivery to her newborn, but followed by the suffix NN.

The interview was followed by the full physical examination of these parturients. At the same time, to assess the nutritional status of parturients, their mid-upper arm circumference (MUAC) was measured. Thus, in accordance with the technical files of National Nutrition program of the DRC [10], a MUAC ≥ 22 cm was considered as a normal nutritional status, a MUAC entry ≥ 21 to <22 cm as mild malnutrition and a MUAC < 21 cm was considered as severe malnutrition.

Immediately after delivery, nurses or midwives thoroughly examined completely newborns while weighing them using the SH-8008 electronic baby scale. Referring to the WHO definition of low birth weight [11], a birth weight lower than 2500 gr at birth was considered as low birth weight (LBW). The laboratory technicians of the study performed the thick drop smear (TDS) in placental prints and umbilical cord blood.

### 2.5. Laboratory Investigations

#### 2.5.1. Training

The laboratory technicians had been briefed on techniques of collecting peripheral blood, blood from placental prints, and umbilical cord blood from the newborn and the techniques of spreading, preparing, and conveying samples according to the guidelines of the NMCP. They have also been trained on testing HIV/AIDS and prevention of other sexually transmitted diseases as recommended by the National AIDS Control Program (NACP).

#### 2.5.2. HIV Test

Upon admission of the parturients to the labor room, after obtaining their informed consent, the laboratory technicians collected 2 ml of venous blood in an EDTA tube. They then performed rapid HIV test with the rapid test Determine assay HIV. Positive cases were referred to the provincial laboratory of the NACP in Kisangani for confirmation of the diagnosis, whereby all HIV-positive mothers were subjected with their newborns to antiretroviral treatment according to the DRC policy related to HIV prevention and care [12].

#### 2.5.3. Malaria Diagnosis

Immediately after delivery, after clamping and cleaning the umbilical cord, laboratory technicians took 1 ml of blood by puncture of the umbilical vein and immediately spread it on a slide. Then, to perform the placental print, they cleaned the placenta superficially, incised its maternal surface, and applied a blade on the incised placental surface. These slides, umbilical blood smears and placental print, were stained for 10 minutes with 10% diluted Giemsa solution, before being sent to the Provincial Public Health Laboratory (PPHL) for microscope reading at the objective 100x. The diagnosis of malaria parasitaemia was considered when trophozoites or other plasmodium development stages were present in the sample. Thus, placental malaria was defined as the presence of malaria parasitaemia in the placental print, whereas congenital malaria was defined as the presence of malaria parasitaemia in umbilical blood smear among newborns who were born from mothers with placental malaria.

For the quality control of the results obtained at the PPHL, 10% of random positive samples and 10% of random negative samples were transferred and analyzed at the National Institute of Biomedical Research in Kinshasa; and the concordance rate, by calculating the Kappa index, was 85% for the same analyses.

### 2.6. Statistical Analysis

The collected data was encoded in Excel and imported for analysis using STATCALC software Epi info 7.2.2.6. We described the sample by calculating the mean, the frequency, and the percentage, as well as the averages and their standard deviations.

To compare proportions, we calculated Pearson's chi squared or fisher's exact test at the level of significance <0.05.

To measure the strength of the association, the odds ratio (OR) and its 95% confidence interval (CI) were determined.

### 2.7. Ethical Considerations

Before conducting this study, we submitted the protocol to the ethics committee of Kisangani University and obtained its approval (Approval No. UNIKIS/CER/006/2018). To participate in the study, mothers of newborns had to sign the informed consent form. All newborns and mothers in whom the TDS was positive were subjected to antimalarial treatment according to the PNLP-recommended regimen in the DRC.

## 3. Results

### 3.1. General Characteristics of Newborns' Mothers (*N* = 1248)

The mean age of newborns' mothers was 25.40 ± 6.24 years; 170 newborns' mothers (13.62%) were under the age of 19; 24.36% were primiparous; 95.03% of them used the ITNs; 95.27% used IPTp-sp; the proportion of respondents with positive HIV serology was 3.37%; 186 (14.90%) had placental malaria.

### 3.2. Characteristics of Newborns' Mothers Who Had Placental Parasitaemia

We notice that 128 newborns' mothers who had placental malaria (68.82%) were aged 19 to 34 while 52 (27.96%) were under 19; 52.69% of them were primiparous; 174 (93.55%) followed at least one ANC session and took at least one IPTp-sp dose; 168 (90.323%) were using ITNs; 5.38% were HIV positive; the mean of their MUAC was 22.1812 ± 1.4147; and 90.86% of them had a normal nutritional status (MUAC ≥ 22 cm) (Table 1).

### 3.3. Prevalence of Congenital Malaria

In 186 TDS performed in umbilical cord blood samples of newborns from mothers with placental malaria, 26 were positive, as a prevalence of congenital malaria of 13.98%.

### 3.4. Profile of Mothers of Newborns Who Have Contracted Congenital Malaria

In Table 2, we described the profile of mothers of newborns who have contracted congenital malaria.

We found that 69.23% of newborns who have contracted congenital malaria were born from mothers aged 18 to 34, 53.85% from primiparous mothers, 92.31% from mothers who took IPTp-sp; all newborns who have contracted congenital malaria (100%) were born from mothers using ITNs and 7.69% from HIV-positive mothers (Table 2).

### 3.5. Perinatal Complications of Congenital Malaria

In Table 3, we described the complications recorded in the newborns who have contracted congenital malaria.

We found that 76% of newborns who have contracted congenital malaria had low birth weight. We recorded perinatal mortality in 7.69% of cases (Table 3).

### 3.6. Relationship between Taking IPTp-sp and the Outcome of Pregnancy

In Table 4, we determined the relationship between IPTp-sp and the outcome of pregnancy among parturients who had placental malaria.

While reading this table, we notice that, among parturients with placental malaria, taking IPTp-sp had no effect on congenital malaria (FE = 0.5218; OR: 0.8, 95% CI: 0.1651-3.8769) and LBW (FE = 0.3675; OR: 1.2308, 95% CI: 0.0037-0.1464); however, it seemed to have protective effect against perinatal mortality (FE = 0.0001; OR: 0.0233, 95% CI: 0.0037-0.1464) (Table 4).

## 4. Discussion

### 4.1. Prevalence of Congenital Malaria

In this study, the prevalence of congenital malaria in Kisangani was 13.98% (95%CI = 9.34‐19.81). The prevalence of congenital malaria varies from region to region or from a study to another, and it is often influenced by the methodology used to define the case. In this study, the population consisted of newborns of mothers with placental parasitaemia and congenital malaria was defined by positivity of the umbilical cord blood of these newborns.

The prevalence of congenital malaria that we found in Kisangani is higher than that found by Piñeros-Jiménez et al. [13] in Uraba in Colombia (2.5%), by Enweronu-Laryea et al. [14] among Ghanaian newborns (2.2%), by Mosha et al. [15] in Morogoro in Tanzania (0.5%; 95% CI: 0.0-1.5%), by Mwaniki et al. [16] in Kenya (0.35%), that reported by Fischer's investigation in Africa [17] (7%), by Falade et al. [18] in Ibadan (2.6%), in Ilorin (3.4%), and in Kaduna (8.5%). In contrast to us, these different authors had identified newborns without taking into account placental malaria parasitaemia. In addition to this reason, for Mwaniki et al. [16], congenital malaria was defined as the detection in newborns of asexual intraerythrocytic forms of plasmodium species in the first 7 days after birth.

Our result is lower than that of Bah et al. [19] who found a prevalence of congenital malaria of 15.57% in Guinea. In addition to the TDS in cord blood and placenta, like in ours, these authors also took into account the TDS in the heel to define the case of congenital malaria.

The prevalence of our study is also less than the one found by Lehner and Andrews [20] in Guinea (14.6%) and Obiajunwa et al. [21] in Nigeria (54.2%). In Benin, Sagbo et al. [22] found 57% of congenital malaria in newborns and 17.08% in those whose mothers had malaria during pregnancy. To diagnose congenital malaria, Sagbo et al. [22] had considered the TDS and the malaria rapid test on the umbilical cord or peripheral blood of the newborn. Obiajunwa et al. [21] found a strong association between placental malaria and cord parasitaemia. Indeed, the prevalence of placental malaria parasitaemia was very high in their study. The high prevalence of congenital malaria recorded by us, as by these authors, would also be related to the fact that Kisangani is a stable malaria transmission zone.

### 4.2. Profile of Mothers of Newborns Who Have Contracted Congenital Malaria

We found that 69.23% of newborns who have contracted congenital malaria were born from mothers aged 18 to 34, 53.85% from primiparous mothers, 92.31% from mothers who took IPTp-sp; all newborns (100%) were born from mothers using ITNs and 7.69% from HIV-positive mothers.

As for age, our result is similar to that found by Noudamadjo et al. [23] who found that the age of newborns' mothers with congenital malaria was less than 30 years in 78.9% of cases and between 20 and 30 years in 66.7% of cases. Bah et al. [19] found an average age of newborns' mothers with congenital malaria of 26 ± 14 years with prevalence in the age group of 20-35 years (4.7%) while Agossou et al. [24] had found an average age of 27.3 ± 6 years with extremes of 15 to 42 years. Vanga-Bosson et al. [25] found a median of 17 years.

Compared to parity, in our research, 53.85% of newborns with congenital malaria were born from primiparous mothers. This prevalence is lower than that reported by Agossou et al. [24] (62.9%) and the one found by Vanga-Bosson et al. [25] in Ivory Coast (100%).

In our research, 92.31% of newborns with congenital malaria were born from mothers who completed ANC. This result does not agree with Bah et al. [19] who had diagnosed congenital malaria in 7.3% of newborns of mothers with at least 4 CPN.

We found that 92.31% of newborns who had had congenital malaria were born from mothers who took IPTp-sp. Fischer [17] found a prevalence of congenital malaria of 6.1% in newborns of mothers who took IPTp-sp. Vanga-Bosson et al. [25] found a prevalence of 4.7% (95% CI: 1.3-11.6%).

In our research, 7.69% of our respondents were born to HIV-positive mothers. In their study, Vanga-Bosson et al. [25] found that no newborn mother with congenital malaria was infected with HIV.

In a study conducted on the prevalence of gestational malaria in Kisangani, Labama et al. [26] found that the prevalence of gestational malaria was very high in Kisangani and was associated with younger age, primiparity and HIV and that monitoring ANC and taking IPTp-sp did not protect Kisangani's pregnant women from malaria. We believe that the findings made in that study justify our results.

### 4.3. Perinatal Complications of Congenital Malaria

In our study, 76% of newborns with congenital malaria had low birth weight. We recorded perinatal mortality in 7.69% of cases.

In a study in Ghanaian newborns, Enweronu-Laryea et al. [14] found that 55% of newborns with congenital malaria had a low birth weight.

These complications would be due to uteroplacental hypoperfusion. In malaria-endemic areas such as Kisangani, in case of malaria infection, plasmodiums are sequestered in the placenta [8]. This would be the cause of the decrease in uteroplacental perfusion, which would lead to intrauterine growth retardation, causing low birth weight. Decreased uteroplacental perfusion would also lead to fetal distress that may lead to perinatal mortality.

### 4.4. Relationship between Taking IPTp-sp and the Outcome of Pregnancy

Taking IPTp-sp had no effect on congenital malaria (FE = 0.5218; OR: 0.8, 95% CI: 0.1651-3.8769) and LBW (FE = 0.3675; OR: 1.2308, 95% CI: 0.0037-0.1464); however, it seemed to have protective effect against perinatal mortality (FE = 0.0001; OR: 0.0233, 95% CI: 0.0037-0.1464).

Taking IPTp-sp had no effect on congenital malaria and low birth weight because of the resistance of plasmodium to Sulfadoxine-Pyrimethamine, reported in DRC [27]. It was reported in a study conducted in Kisangani that IPTp-sp did not have an effect on the birth weight [28].

Because of the size of our sample with a small proportion of perinatal mortality, it is difficult to comment on the apparent protective effect of IPTp-sp against perinatal mortality. Indeed, the noneffect on congenital malaria and low birth weight, in a context of plasmodium resistance to Sulfadoxine-Pyrimethamine, would cause IPTp-sp to have no protective effect on perinatal mortality. This calls for a more extended study to clarify these facts.

## 5. Conclusion

Congenital malaria remains an important problem in areas of stable malaria transmission such as Kisangani. Its prevalence is 13.98% and it is grafted with major perinatal complications, particularly the low birth weight of newborns and perinatal mortality. We recommend an extended study to clarify the relationship between the outcome of pregnancy and the intermittent preventive treatment in pregnancy with Sulfadoxine-Pyrimethamine.

## Figures and Tables

**Table 1:** Characteristics of newborns' mothers who had placental parasitaemia (*N* = 186).

Newborns' mothers	*n* (%)	95% CI
Age (years)		
<19	52 (27.96)	21.64-34.99
19-34	128 (68.82)	61.63-75.39
≥35	6 (3.23)	1.19-6.89
Level of education		
Illiterate	8 (7.20)	1.87-8.30
Primary	32 (4.30)	10.32-26.89
Secondary	118 (63.44)	56.08-70.37
Higher and university	28 (15.05)	10.24-21.02
Profession		
Student	16 (8.89)	5.17-14.03
Housewife	154 (85.56)	79.56-90.34
Employee	6 (3.33)	1.23-7.11
Informal sector	4 (2.22)	0.61-5.59
Residence		
Rural	32 (17.20)	12.08-23.41
Urban	154 (82.80)	76.59-87.92
Parity		
1	98 (52.69)	45.25-60.04
2 and more	88 (47.31)	39.96-54.75
ANC visits		
0	12 (6.45)	3.38-11.00
1	28 (15.05)	10.24-21.02
2	18 (9.68)	5.84-14.86
3	76 (40.86)	33.72-48.29
4	38 (20.43)	14.88-26.95
5	14 (7.53)	4.18-12.31
IPTp-sp doses		
0	12 (6.45)	3.38-11.00
1	22 (11.83)	7.56-17.36
2	23 (12.37)	8.00-17.97
3	129 (69.35)	62.19-75.89
Use of ITNs		
No	18 (9.68)	5.84-14.86
Yes	168 (90.32)	85.14-94.16
HIV serology		
Negative	176 (94.62)	90.34-97.36
Positive	10 (5.38)	2.61-9.66
MUAC (cm) (mean: 22.1812 ± 1.4147)		
≥22	169 (90.86%)	85.77-94.59
≥21 to <22	16 (8.60%)	5.00-13.59
<21	1 (0.54%)	0.01-2.96

**Table 2:** Profile of mothers of newborns who have contracted congenital malaria (*N* = 26).

Mothers	*n* (%)	95% CI
Age (years)		
<19	8 (30.77)	14.33-51.79
19-34	18 (69.23)	48.21-85.67
≥35	0 (0.00)	—
Parity		
1	14 (53.85)	33.37-73.41
2 and more	12 (46.15)	26.59-66.63
Taking IPTp-sp		
No	2 (7.69)	0.95-25.13
Yes	24 (92.31)	74.87-99.05
Maternal fever		
No	24 (92.31)	74.87-99.05
Yes	2 (7.69)	0.95-25.13
Using of ITNs		
No	0 (0.00)	—
Yes	26 (100)	86.77-100
HIV serology		
Negative	24 (92.31)	74.87-99.05
Positive	2 (7.69)	0.95-25.13

**Table 3:** Perinatal complications of congenital malaria (*N* = 26).

Perinatal complications	*n* (%)	95% CI
Low birth weight		
No	6 (23.08)	8.97-43.65
Yes	20 (76.92)	56.35-91.03
Perinatal mortality		
No	24 (92.31)	74.87-99.05
Yes	2 (7.69)	0.95-25.13

**Table 4:** Relationship between taking IPTp-sp and the outcome of pregnancy (*N* = 186).

Taking IPTp-sp	Congenital malaria		*p* value	OR (95% CI)
	Yes	No		
No	2 (16.67%)	10 (83.33%)	0.5218^∗^	1.25 (0.2579-6.0576)
Yes	24 (13.79%)	150 (86.21%)		1

	Perinatal mortality			
	Yes	No		
No	4 (3.23%)	8 (86.77%)	0.0001^∗^	0.0233 (0.0037-0.1464)
Yes	2 (2.19%)	172 (97.81%)		1

	LBW			
	Yes	No		
No	6 (50.00%)	6 (50.00%)	0.3675^∗^	1.2308 (0.0037-0.1464)
Yes	96 (55.17%)	78 (44.83%)		1

^∗^FE.

## Data Availability

All data used to support the findings of this study are available from the corresponding author upon request.

## Abbreviations

ANC: Antenatal care
CI: Confidence interval
DRC: Democratic Republic of Congo
FE: Fisher exact
IPTp-sp: Intermittent preventive treatment in pregnancy with Sulfadoxine-Pyrimethamine
ITNs: Insecticide-treated mosquito nets
LBW: Low birth weight
MUAC: Mid-upper arm circumference
NACP: National AIDS Control Program
NMCP: National Malaria Control Program
OR: Odds ratio
PPHL: Provincial Public Health Laboratory
TDS: Thick drop smear
WHO: World Health Organization.
